# GeneWiz browser: An Interactive Tool for Visualizing Sequenced Chromosomes

**DOI:** 10.4056/sigs.28177

**Published:** 2009-09-25

**Authors:** Peter F. Hallin, Hans-Henrik Stærfeldt, Eva Rotenberg, Tim T. Binnewies, Craig J. Benham, David W. Ussery

**Affiliations:** 1 Center for Biological Sequence Analysis, Department of Systems Biology, The Technical University of Denmark, 2800 Kgs. Lyngby, Denmark.; 2 Lersoe Parkalle 37, 2TV, 2100 Copenhagen, Denmark; 3 Roche Diagnostics Ltd., CH-6343 Rotkreuz, Switzerland; 4 UC Davis Genome Center, University of California, Davis, California, U.S.A.

## Abstract

We present an interactive web application for visualizing genomic data of prokaryotic chromosomes. The tool (GeneWiz browser) allows users to carry out various analyses such as mapping alignments of homologous genes to other genomes, mapping of short sequencing reads to a reference chromosome, and calculating DNA properties such as curvature or stacking energy along the chromosome. The GeneWiz browser produces an interactive graphic that enables zooming from a global scale down to single nucleotides, without changing the size of the plot. Its ability to disproportionally zoom provides optimal readability and increased functionality compared to other browsers. The tool allows the user to select the display of various genomic features, color setting and data ranges. Custom numerical data can be added to the plot allowing, for example, visualization of gene expression and regulation data. Further, standard atlases are pre-generated for all prokaryotic genomes available in GenBank, providing a fast overview of all available genomes, including recently deposited genome sequences. The tool is available online from http://www.cbs.dtu.dk/services/gwBrowser. Supplemental material including interactive atlases is available online at http://www.cbs.dtu.dk/services/gwBrowser/suppl/.

## Introduction

The development of fast and inexpensive genome sequencing technologies has led to the generation of vast amounts of genomic information. As genomic sequencing becomes both more powerful and affordable, the handling and analysis of the generated data produces novel challenges and shifts the focus away from the discovery process towards technical considerations of handling, storing and analyzing sequence data. An important step when exploring a new genome is to compare it to existing sequences, in order to identify both novel and conserved features. Many automated computational methods are available that attempt to derive protein function from sequence [1-3]. In a metagenomic study by Harrington and co-workers it was estimated that 76% of the examined protein coding genes could be assigned a function. However, to assess predictions for individual genes the visualization remains critical to provide the biologist with an overview of the genomic context. Are genes of interest situated in clusters? In operons? How are they regulated? How does their DNA base composition compare with that of the rest of the genome? In order to display such features both on a genome scale and in close-up down to the level of nucleotides, we developed the GeneWiz browser which is based on the ‘Genome Atlas’ concept [4,5]. This tool can also display local DNA structural properties, so that regulatory or repeat regions can easily be identified and interpreted in a chromosomal context.

During development of the GeneWiz browser, it became apparent that novel sequencing technology creates a further demand. The current generation of sequencing instruments utilizes primed synthesis in flow cells to simultaneously obtain the sequences of millions of different DNA templates, an approach that changed the field of DNA sequencing [6,7]. Flow sequencing, also known as sequencing by synthesis (SBS) on a solid surface, tracks nucleotides as they are added to a growing DNA strand [8]. SBS is used by high-throughput sequencing systems which have become commercially available in the past two years. Examples include the sequencer GS Titanium (commercialized by 454/Roche); Genome Analyser GA-II (Solexa/Illumina); and SOLiD™ 3 system (Applied Biosystems).

These developments have increased the speed of sequencing while significantly reducing its cost [9,10]. This much higher throughput provides greater coverage, but at the cost of much shorter read-lengths: from 50 bases with SOLiD 3 to 75 bases with Illumina GA II. Even reads of 500 bases obtained with the 454-Titanium are still shorter than read lengths typically obtained using the Sanger method [9,11]. The output from modern high-through sequencing equipment challenges the assembly software by generating shorter and ambiguous reads. Processing of this flood of sequence data has rapidly become a bottleneck, and developing the necessary skills and tools will most likely be a driving factor in the execution of second-generation sequencing [12]. As a first step in this development, it needs to be determined to what extent assembly of short-read sequences can be trusted, an assessment for which the GeneWiz browser can also be used.

## Methods

Our method of visualization is based on color-encoded lanes to display numerical information on a genome atlas similar to GeneWiz [4,5]. The color encoding can be done either using a linear scale with a fixed minimum and maximum range, or a dynamic scale of standard deviations. Using the latter, color intensity decreases as data approach average values, thereby emphasizing regions of significant variation. The web interface is divided into four optional sections, to address various biological viewpoints of chromosomes: 1) DNA properties 2) Mapping of homologous genes by BLAST 3) Mapping of short sequencing reads 4) Custom lanes such as Single Nucleotide Polymorphism (SNP) or microarray data. The output of each method is a numerical vector of length corresponding to that of the reference sequence, and the methods used for this construction are described in detail below.

### Read quality assessment

Gene duplications, rRNA operons and other repetitive chromosomal regions are known to cause difficulties during the assembly of short reads [13]. To assess the degree of ambiguity of sequencing reads, a method was developed that derives the uniqueness of all reads, accounting for both the read quality and the match to the reference genome.

**Figure 1:**
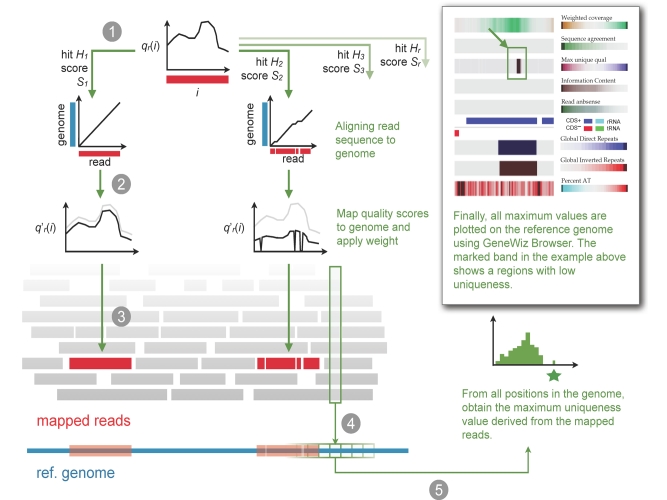
**|** Mapping reads to a reference genome accounting for uniqueness. In step 1, each read is aligned against the reference genome. In the second step, the quality of each read is weighted according to the uniqueness of the hit. A read giving rise to two hits *S_1_* and *S_2_* in the reference genome will be weighted proportionally with the relative alignment scores; if scores are identical, the mapping of *S_1_* and *S_2_* will be applied a weight of *w*=0.5 (see equation below). Step 3 maps the weighted qualities back to the reference genome so that each genomic position contains an array of weighted qualities. Once all reads are mapped, in step 4 only the maximum weighted quality value is kept and, step 5, the maximum weighted quality scores are color coded to reveal regions of low uniqueness.

Sequence reads from Illumina and 454 are reported with base qualities: a per-nucleotide measure that denotes the credibility of the base calls. A method was derived which condenses these qualities into values per position in the reference genome and calculates the following information: uniqueness-weighted quality, information content, sequence agreement, and repeat-weighted coverage, (see methods). These estimates provide a preliminary overview of regions that may appear problematic to assemble. In general, low uniqueness is found in the gaps between the assembled contigs generated by the default assembly tools from a given sequence dataset, as will be demonstrated below. A high score of uniqueness-weighted quality indicates that the base is uniquely identified by a read and that it has a high base quality in that read. The approach is illustrated in Figure 1.

From the mapping, five different parameters were calculate which together summarizes the trustworthiness of the reads given the assembly:

**Weighted coverage** Under the assumption that all reads would map only once (*H_r_*=1), the coverage *c*(*i*) can be calculated as the number of alignments *R* mapped at position *i*. A weighted coverage *c*’(*i*)=*w_r,h_* (see equation below) is used to correct for higher coverage artificially introduced by repeats:


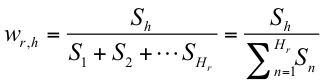


**Figure 2:**
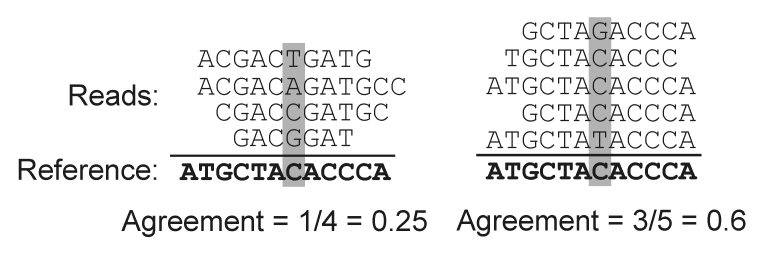
| Agreement between reads and reference sequence.

**Sequence agreement** This denotes the frequency of the reference nucleotide among the reads mapped at a given position and reveals the extent of agreement between the reads and the reference genome, as shown in Figure 2.

**Uniqueness-weighted quality** This measure corresponds to the base qualities obtained from the reads that are mapped to the reference genome, weighted by the uniqueness of the read. Consider read *r*, which has a quality profile , where *i* is the position in the read. The read is aligned to the reference genome by BLAST, and all *H_r_* hits are included, when the following criteria are met: BLAST score *S_h_* of hit *h* is greater than or equal to *S_0_* (optionally provided by the user), *S_h_* ≥ *S_1_* ⋅ *x* where *S_1_* is the score of the first/best hit, *x ∈* [0;1] is a constant provided by the user, and the *E*-value is equal to or less than a threshold specified by the user. The following formula is used to derive the weighted quality:


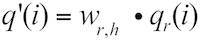


From all the *q’_r_*(*i*) values obtained at each position in the genome, the maximum uniqueness-weighted quality is chosen when all reads have been mapped.

**Information content** provides a number in bits of information [14] representing to what degree the reads agree: zero bits means equal distribution of A, T, G and C at a given position and 2 bits means complete conservation of a single base.


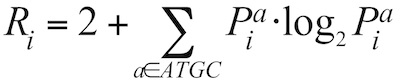


The value is plotted on a color scale whereby low information (random distribution, least expected) is given in dark colors, and high information (high conservation, most expected) as light or neutral color. This measure may be useful for visualizing single nucleotide polymorphisms.

**Read absence.** A boolean where ‘one’ indicates complete absence of aligned reads.

### Visualization of whole-genome homology

The BLASTatlas method [15] derives a map of per-nucleotide numbers on a reference genome to visualize the matches in the alignment between the reference genome and a query. The query can constitute any number of genomic contigs, scaffolds, full genomes, or collections thereof. This provides a method to identify regions of a reference genome that are conserved throughout multiple samples, as well as those that are unique. The BLASTatlas method is integrated into the GeneWiz browser software to facilitate a user-friendly interface. According to the BLAST algorithm chosen, DNA or protein sequences of the reference are aligned with the best match in the query (using either blastp, blastn, tblastn, or blastx). The alignment is then mapped back to the reference genome. A match adds a 'one' whereas a mismatch adds a 'zero' at each position along the chromosome. These ones and zeros translate into smooth color zones due to binning

### DNA properties and DNA destabilization

**Figure 3:**
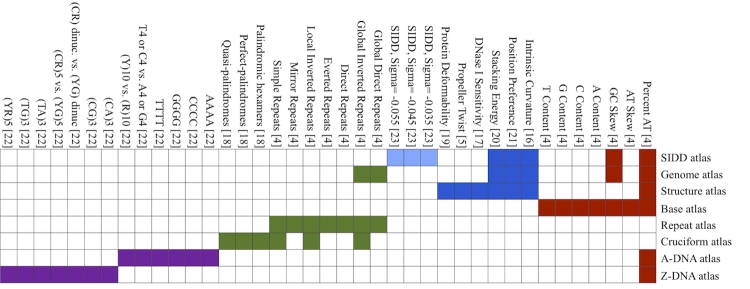
Configuration and references for pre-defined groups of DNA sequence- and structural properties: Genome-, Base-, Structure-, Cruciform-, A-DNA-, Z-DNA-, Repeat-, and SIDD-atlas.

Through the web interface it is currently possible to select from 36 different nucleotide composition and DNA structural properties [4, 5, 16-22]. In addition to this, calculations of so-called SIDD energy estimates are provided, offering an approximation of promoter regions. This method estimates the free energy required to open the DNA helix, calculated at the three different superhelix densities σ = -0.035, -0.044, -0.055, using the SIDD algorithm [23]. All of these parameters can be applied in any combination to any of the prokaryotic genomes available from the web interface, or to a custom sequence provided by the user. Alternatively, the parameters may be applied as collections forming 8 standard atlases: Genome-, Base-, Structure-, Cruciform-, A-DNA-, Z-DNA-, the Repeat-atlas, and finally the SIDD atlas, which is introduced in this manuscript (Figure 3).

### Custom data

A designated section of the GeneWiz browser is assigned for custom data. It allows the user to provide a per-nucleotide list of numerical values along with a desired color and data range. Although not presented here, this allows for visualization of additional information such as microarray data that has been pre-processed by the user, by mapping gene expression, regulation change, or *p-*values back to genomic coordinates. In addition to the main genome annotation covering CDSs, tRNAs, and rRNAs, the user may specify miscellaneous and pseudo-gene annotations separately. A button allows the query of selected reference genomes against a replicate of pseudogenes.org [24]. Other annotations of possible pseudogenes can be added, such as GenePRIMP output (geneprimp.jgi-psf.org/).

### Dynamic visualization

The GeneWiz browser allows dynamic disproportional zooming, meaning that zooming occurs nearly instantly when requested by the user, by redrawing all the components like tracks, legends, marks and text for every view. This allows the browser to scale the plot to make use of the entire plotting area, by not rescaling all parts of the plot equally. For example, zooming 10x will stretch a data lane 10 in genome position axis, however the lane height and distance to the neighbor lane will remain constant. The dynamic nature of the GeneWiz browser requires pre-binning of data for each zoom level, all of which are stored on a central server; for improved efficiency only data requested by the user are sent. The approach to store per-nucleotide information as table records in a database (e.g. MySQL) has proved unfeasible, as the number of records per genome exceeds millions, and the construction of indexes would be very time consuming. Instead, a memory mapping technique was chosen, that allows the server to directly obtain the values from binary files when provided with the zoom window and level, for any chromosome in the database. (Examples are provided as supplemental data, http://www.cbs.dtu.dk/services/gwBrowser/suppl/).

The client is written as a JavaApplet, that obtains the data remotely from the server (http://ws.cbs.dtu.dk/cgi-bin/gwBrowser-0.91/server.cgi). The browser server is written in Perl/CGI, while a compiled *c*-program handles the access to the binary data files. The options currently supported are listed in Table 1.

**Table 1:** GeneWiz Browser server options.

**key**	**value**	**description**
**d**		The unique identifier for the atlas
**ft**		Feature type (e.g. CDS,rRNA,tRNA) when returning annotations
**f**		Data field to return
**b**		Begin of window
**e**		End of window
**l**		Zoom level
**z**		Enable zlib compression of output
**m**	***i***	Return the genome length
	***avg/stddev/*** ***min/max***	Return aggregate data for window/genome
	***d***	Return data values provided field, window and zoom level
	***c***	Return colors provided two or three-step ranges
	***n***	Return nucleotides provided the window
	***a***	Return annotations (used together with option ‘ft’)

These options (Table 1) can be incorporated into a single URL. For example, one could request all numerical data for field f=dnap0, at zoom level l=5, from position b=1 to e=37,473bp, using compression, z=true (http://ws.cbs.dtu.dk/cgi-bin/gwBrowser-0.91/server.cgi?d=AL111168GENOMEatlas&m=d&f=dnap0&b=1&e=37473&l=5&z=true). The field names and their configurations are described in the xml record, which can be downloaded from the web (http://ws.cbs.dtu.dk/cgi-bin/gwBrowser-0.91/fetchxml.cgi?AL111168GENOMEatlas). Further examples are provided in the supplemental data section.

### The GeneWiz browser workflow and data displayed

**Figure 4:**
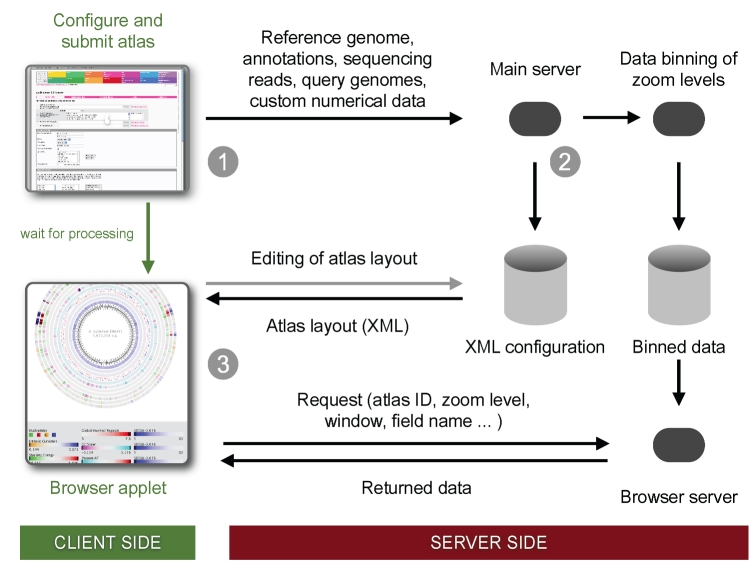
| The dataflow of the GeneWiz browser service. 1) The selected reference genome and the lanes to be included are defined via the web interface. 2) The request is sent to the analysis server that handles the calculations. 3) When the job is finished, the web page redirects to the applet viewer that allows the user to navigate and edit the plot layout.

The GeneWiz browser plots and provides disproportional zooming for data pertaining to features and genes as well as numerical data associated with each nucleotide. The disproportional capability of the GeneWiz browser implies that all components (legends, tracks, marks, *etc*.) are regenerated for every view requested by the user. Figure 4 outlines the GeneWiz browser workflow.

When submitting a job via the web interface, the request is assigned a job identifier, under which all data lanes and configurations are kept. After the job has been processed the user may alter lane order, colors, ranges, and append various types of marks to the plot. The layout of a given browser instance is governed by an XML file, located on the server. When generating the graphical representation of the genome, the client Java program will make requests to the server to acquire aggregated values, such as the averages, standard deviations, minima, and maxima as well as lane data and annotations.

### Premade atlases

The genome sequences stored in the CBS Genome Atlas Database [25] are synchronized with NCBI Entrez genome projects and have been pre-processed for all of the eight standard atlases mentioned above. This allows the user to select from currently 1,636 pre-binned replicons from 864 prokaryotic sequencing projects, searchable by replicon name, GenBank accession number, or organism name (http://www.cbs.dtu.dk/services/gwBrowser/precalc/)

## Results

### Evaluation of re-sequencing quality

**Figure 5:**
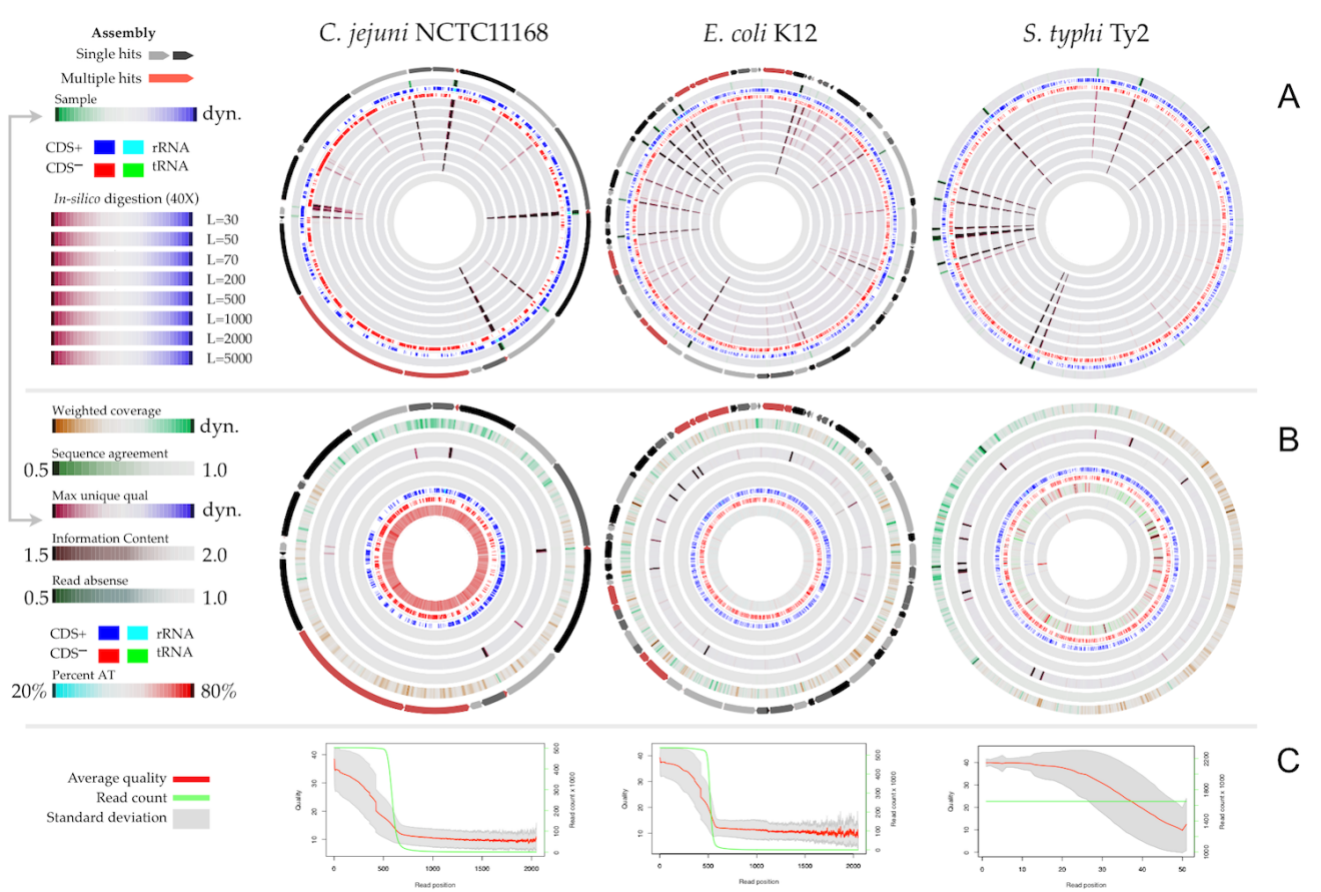
| Panel A: The maximum uniqueness quality is shown for the actual reads (green-to-blue lane) plotted in the outermost lanes, using the published genome as a reference. The following lanes show in-silico digestions at 40X coverage (red-to-blue lane), using read lengths 30, 50, 70, 200, 500, 1,000, 1,000, and 5,000 bases. Panel B shows the weighted coverage, agreement with reference, maximum uniqueness quality, information content, read absence, and AT content. All six plots can be accessed for zooming via the supplemental data section. Panel C displays the read count (green, secondary ordinate) and read quality (red, primary ordinate) as a function of read length. Note that read counts differ within the three datasets, resulting in different scales on the secondary ordinate. For the two 454-Titanium sets (C. jejuni and E. coli K12), an assembly was provided which allows a mapping of contigs to the reference genome. These marks are shown in gray in the perimeter of these plots. Red marks indicate contigs with two or more hits in the reference.

Three re-sequenced bacterial genomes were examined, one genome sequence was generated using the Illumina GA technology, whereas two genome sequences were generated utilizing the 454-Titanium technology (Table 2). The public sequence was selected as reference for mapping the re-sequencing reads using the GeneWiz browser tool. The randomness in fragmentation was estimated by comparing the experimental data with *in-silico* digestions, generated at 40X coverage using read lengths between 30 to 5,000 bp. A good correspondence between the *in-silico* and experimental reads suggests little bias towards certain chromosomal regions (Figure 5, panel A). The assembled contigs provided by 454 (*C. jejuni* and *E. coli*) are mapped to the reference genome using BLAST and annotated in the perimeter of the atlases (two leftmost atlases in Figure 5, panel A+B). The detailed atlas of the experimental data (true reads), are shown in Figure 5, panel B. Panel C shows quality/count of reads plotted as a function of read position. Note that the read quality decreases the further the distance from the beginning of the read.

**Table 2:** Sequencing details of three bacterial genomes, two of which were re-sequenced using 454-Titanium and one with Illumina GA technology.

	***E. coli K12* MG1655**	***C. jejuni* NCTC11168**	***S. typhi* Ty2**
**Strain id**	ATCC: 700926D-5	ATCC: 700819D-5	ftp://ftp.era.ebi.ac.uk ERA000001
**Technology**	454-Titanium	454-Titanium	Illumina GA II
**Read count**	538,784	502,438	1,650,370
**Avg. read length,** **std. dev., and truncation**	522 bp (σ=53) [600 bp]	598nt (σ=75) [600 bp]	51 [35 bp]
**Coverage**	61X	183X	18X
**Genome size**	4,639,675 bp	1,641,481 bp	4,791,961 bp
**Accession and original Reference**	U00096 [26]	AL111168 [27]	AE014613 [28]

### Genome homology: Comparing multiple Burkholderia species

**Figure 6:**
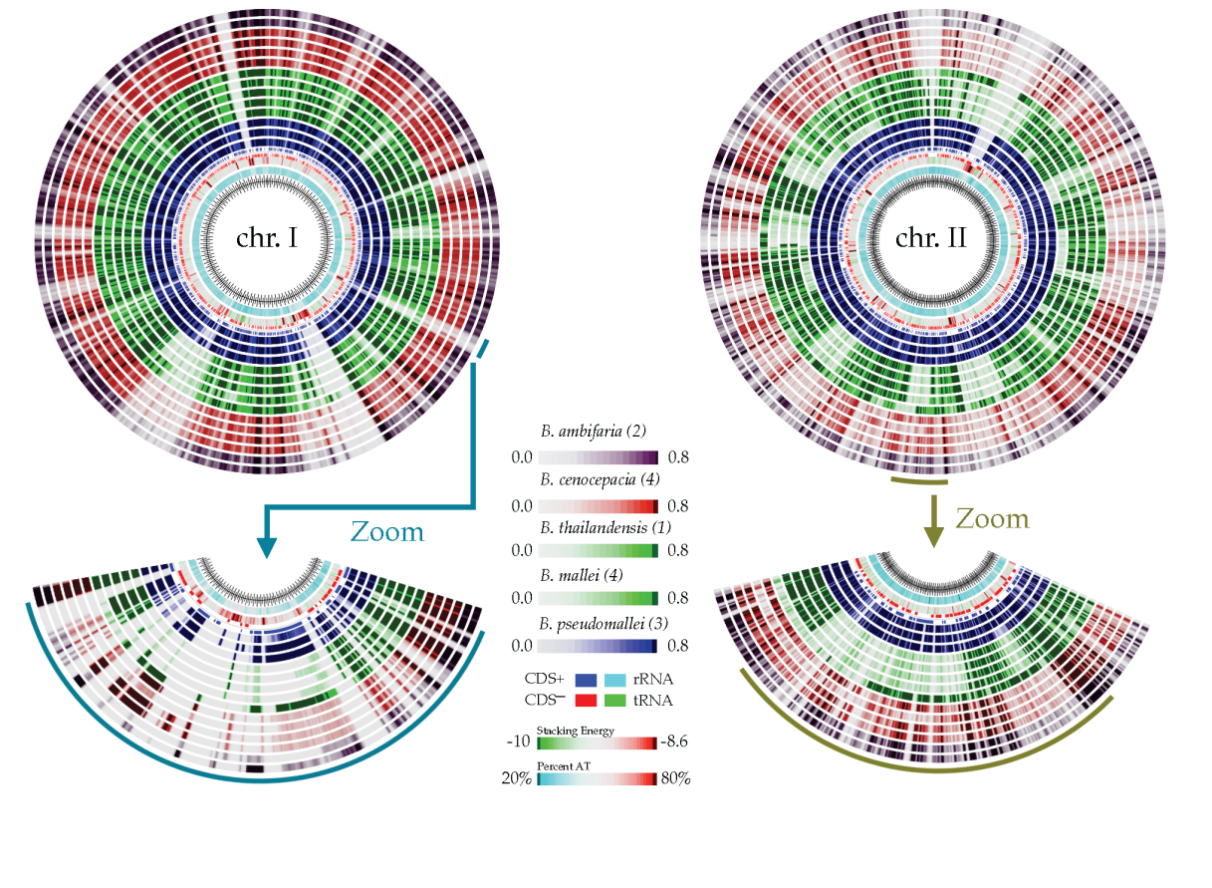
**|** BLASTatlas of *Burkholderia pseudomallei* 1710b chromosomes I+II compared with 14 *Burkholderia* species. Showing from the outermost circles: *B. ambifaria* (2, purple), *B. cenocepacia* (4, red) *B. thailandensis* (1, green) 10774, *B. mallei* (4, green), and *B. pseudomallei* (3, blue). Innermost circles show percent AT, and CG skew. Note, that to allow visual comparison between *B. thailandensis* and *B. mallei*, both species are colored green: the outermost green lane corresponds to the single *B. thailandensis*, whereas the remaining four green lanes are all *B. mallei*. GenBank accession numbers as well as interactive plots are available through the supplemental data section.

A comparative study aimed at mapping for example pathogenic islands or gene losses among different bacterial genomes can benefit from a graphical representation provided by the BLASTatlas method. The genus of *Burkholderia* covers a number of important animal and human pathogens known to cause melioidosis (*B. pseudomallei*) and pulmonary infection in cystic fibrosis (CF) patients (*B. cepacia*), whereas *B. thailandensis*, which is closely related to *B. pseudomallei*, rarely gives rise to diseases in humans [29,30]. Both species of *B. thailandensis* and *B. mallei* display large chromosomal deletions when compared to *B. pseudomallei*. However, the more scattered nature of the gene loss observed in *B. thailandensis* suggests that *B. mallei* evolved from *B. pseudomallei* through the loss of larger regions [31]. These deletions are evident from the atlas shown in Figure 6 where the two chromosomes of *Burkholderia pseudomallei* 1710b are used as BLASTatlas reference in a comparison with 14 publicly available *Burkholderia* genomes (*B. thailandensis* plus all species having two or more strains sequenced, see supplemental data). In addition it is evident that a strong preference of deletion exist for chromosome II. Ong and co-workers report that deletions in chromosome II counts for 70% and 61% of the total gene loss in *B. mallei* and *B. thailandensis,* respectively.

### The SIDD atlas: Annotation of regulatory elements

**Figure 7:**
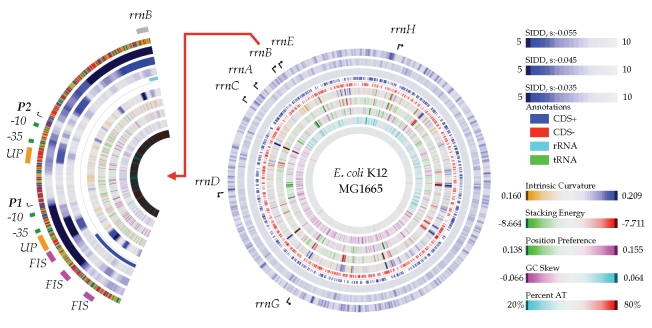
| A zoom upstream of the *E. coli* K12 MG1665 *rrnB* operon. The three outer-most lanes show SIDD at three superhelix densities of sigma=-0.055, -0.045, and -0.035. The lower free energy required to melt the helix can be observed near the UP element of P1, for the SIDD lane at sigma = -0.045. The atlas is available for zooming on the supplemental data section.

The browser application enables the user to append various annotation marks such as transcription start site arrows, gene labels, and boxes. A final example illustrates how these marks can be used to integrate known regulatory elements with DNA properties and gene annotations to draw a more complete picture of a promoter region. The regulatory elements of the *E. coli* K12 MG1665 *rrn* operons [32] have been annotated in a standard SIDD atlas, providing a visualization of the P1/P2 promoter structure (Figure 7). A zoom of the promoter region reveals a strong SIDD site near the predominant P1 promoter approximately 40 bp. upstream of the P1 transcription start site. The transcription factor FIS stimulates transcription at several promoters, and for example the binding of FIS at the *leuV* promoter [33] has been suggested to transmit the superhelical destabilization downstream to the point where the RNAP twists and opens the helix [34]. This model may be valid for the *rrnB* P1 promoter also, as the activity of *leuV* and *rrnB* P1 are comparable [35].

## Discussion

Visualization of the multidimensional information that is represented by a single genome sequence remains complex. An indispensable property of a genome visualization tool is that it must be zoomable, so that information can be interpreted at varying scales. Two recently published methods, the DNAPlotter [36] and the Genome Projector [37], both enable the user to build circular plots of numerical data related to genes as well as graphs of numerical data pertaining to the nucleotides. These tools create static graphics and allows only for proportional zooming, hence making the plot hard to interpret when zooming too deep. Both of these tools allow for visualization of individual genomes, but do not allow easy comparison across multiple genomes. With the ease of new genome sequences becoming available, it is essential to be able to quickly compare other genomes to a reference.

A number of other tools approach genome visualization from different angles: Genome Diagram [38] and Circos [39] are command line programs generating publication quality static images and vector graphics. Although these tools allow comparison of other genomes, are flexible and allow visualization of numerical data, they lack an interactive layer.

The GeneWiz browser described here uses disproportional zooming to overcome this. From a technical perspective, the choice of programming language for writing graphical browsers is of importance. There are obvious advantages of providing platform-independent Java software like that of the GeneWiz browser, but often this is at the cost of performance. Nevertheless, our tool demonstrates the usefulness of a genome browser that relies on interactive, true disproportional zooming to visualize annotated genes and features as well as numerical data provided at single nucleotide resolution. By building a comprehensive tool that is both scalable and flexible, we have shown how different types of genomic data can be integrated into a single, easily navigated graphic that can be annotated further by the user.

## 

### Author contributions

P.F.H. wrote the paper and composed the web interfaces, as well as most parts of the server back end. H.H.S. wrote the c-code of the data binning and retrieval software and contributed to the Java Applet; E.R. wrote the majority of the Java Applet code and formulation of the XML configurations. T.T.B. provided source data and analysis of *C. jejuni* and *E. coli* sequencing reads and C.J.B. assisted writing the paper (paragraphs on SIDD energy). D.W.U. assisted in writing the paper, supervised the project and provided ideas for figures and analysis. All authors have read and made corrections to the manuscript.
